# Author Correction: Interfacial friction enabling ≤20 μm thin free-standing lithium strips for lithium metal batteries

**DOI:** 10.1038/s41467-024-55734-5

**Published:** 2025-01-09

**Authors:** Shaozhen Huang, Zhibin Wu, Bernt Johannessen, Kecheng Long, Piao Qing, Pan He, Xiaobo Ji, Weifeng Wei, Yuejiao Chen, Libao Chen

**Affiliations:** 1 State Key Laboratory of Powder Metallurgy, Central South University, Changsha, Hunan China; 2 Australian Synchrotron, ANSTO, Clayton, VIC 3168 Australia; 3 College of Chemistry and Chemical Engineering, Central South University, Changsha, Hunan China

**Keywords:** Batteries, Energy

**Correction to:** *Nature Communications* 10.1038/s41467-023-41514-0; published online 14 September 2023

The original version of this Article contained a typographical error in the affiliation of the co-author, Bernt Johannessen. “Australian Synchrotron, Clayton, VIC, Australia” should have read “Australian Synchrotron, ANSTO, Clayton VIC 3168, Australia”. The corresponding affiliation in author information has been updated in both the PDF and HTML versions of the Article.

