# Supplementary material for: Interfacial friction enabling ≤ 20 μm thin free-standing lithium strips for lithium metal batteries
Source: Nat Commun. 2023 Sep 14;14:5678. doi: 10.1038/s41467-023-41514-0 (PMC10502130; doi:10.1038/s41467-023-41514-0)
Supplement: Supplementary file 1 — Supplementary Information File [file 41467_2023_41514_MOESM1_ESM.pdf]

Supplementary Information

Interfacial Friction Enabling $\leqslant$ 20 μm Thin Free-standing Lithium Strips for Lithium Metal Batteries

*Shaozhen Huang, Zhibin Wu, Bernt Johannessen, Kecheng Long, Piao Qing, Pan He, Xiaobo Ji, Weifeng Wei, Yuejiao Chen and Libao Chen**

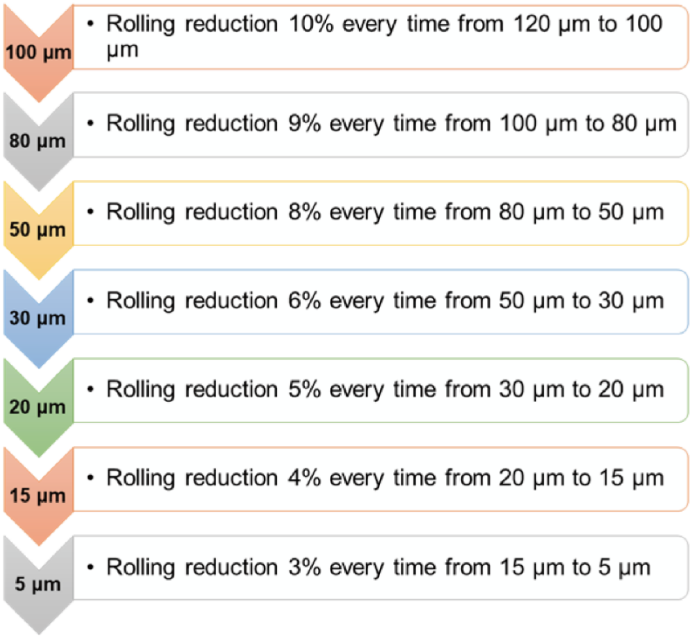

Figure S1. Schematic diagram of the rolling process for different thicknesses of lithium strips.

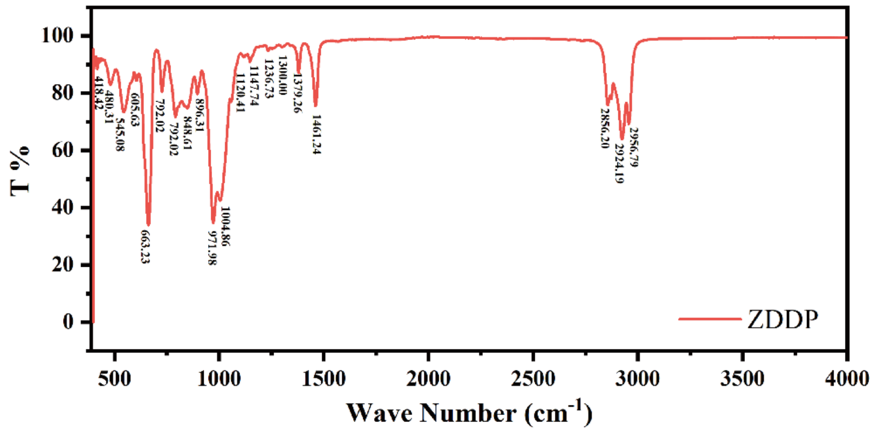

Figure S2. Image of infrared spectroscopy of ZDDP.

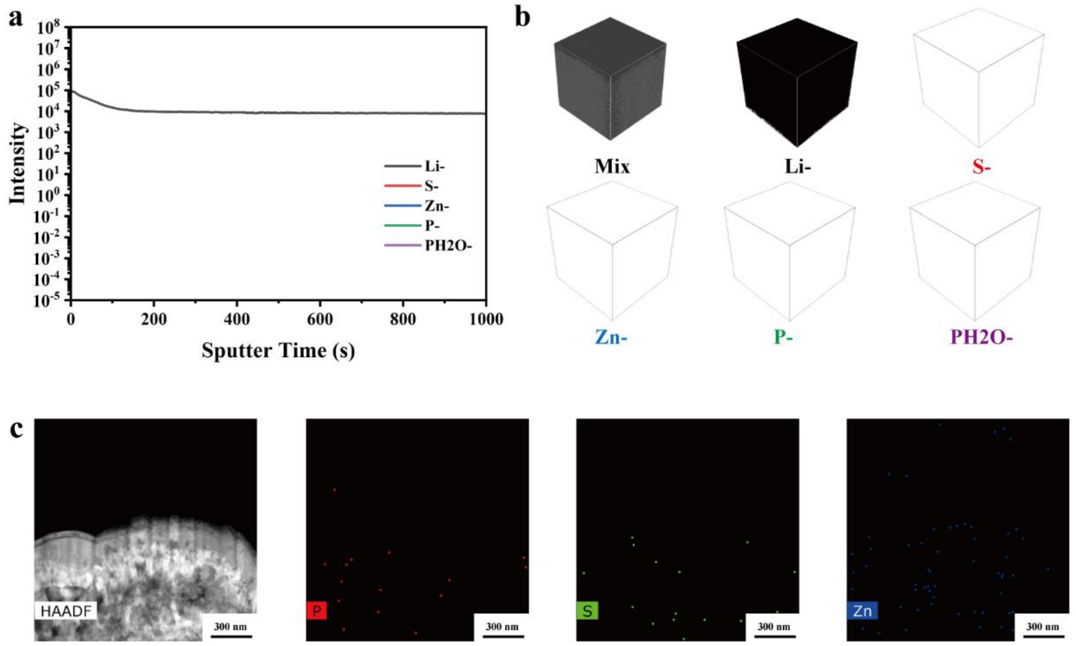

Figure S3. (a) The TOF-SIMS profiles of different atom counts with the depth increasing on bare Li. (b) 3D structure views for TOF-SIMS depth sputtering on the surface of bare Li. (c) Photographs of the Li surface layer and elemental distribution generated by bare Li cross-sectional observation with cryo-TEM.

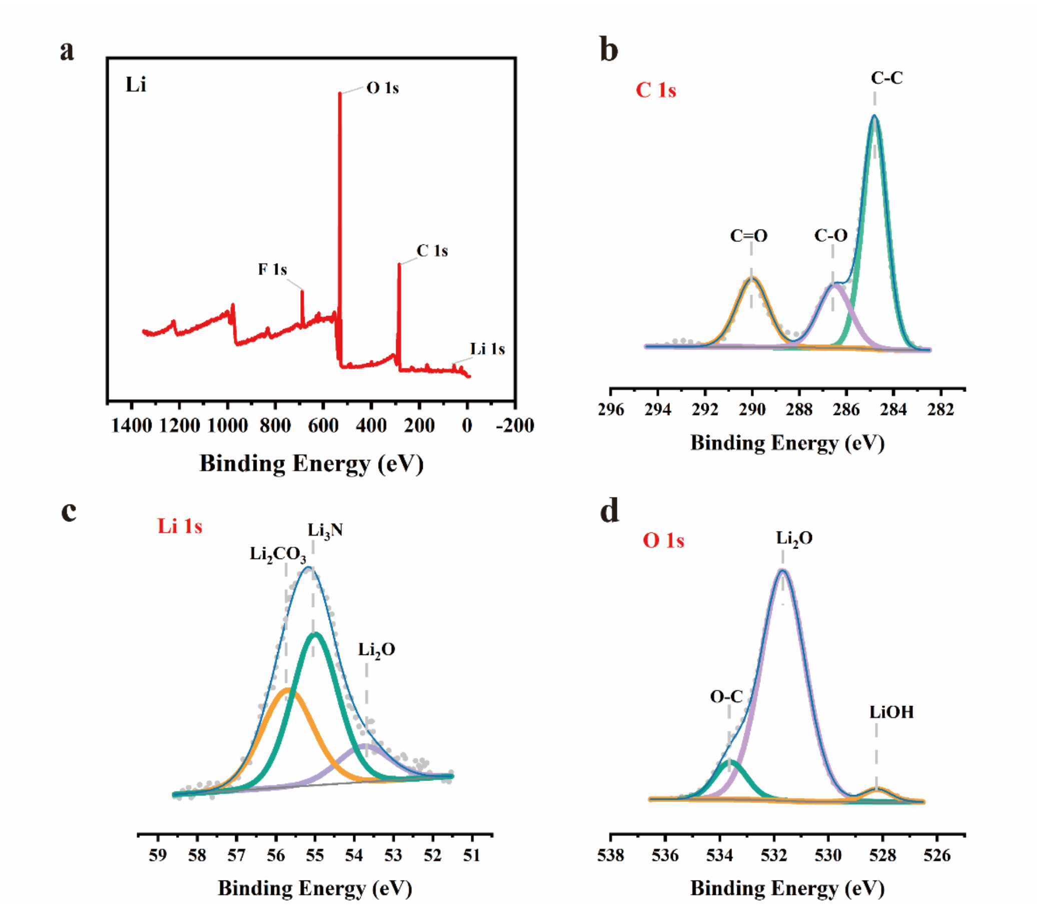

Figure S4. XPS spectra of Li electrode for the initial. (a) Survey, (b) *C 1s* spectra, (c) *Li 1s* spectra, (d) *O 1s* spectra.

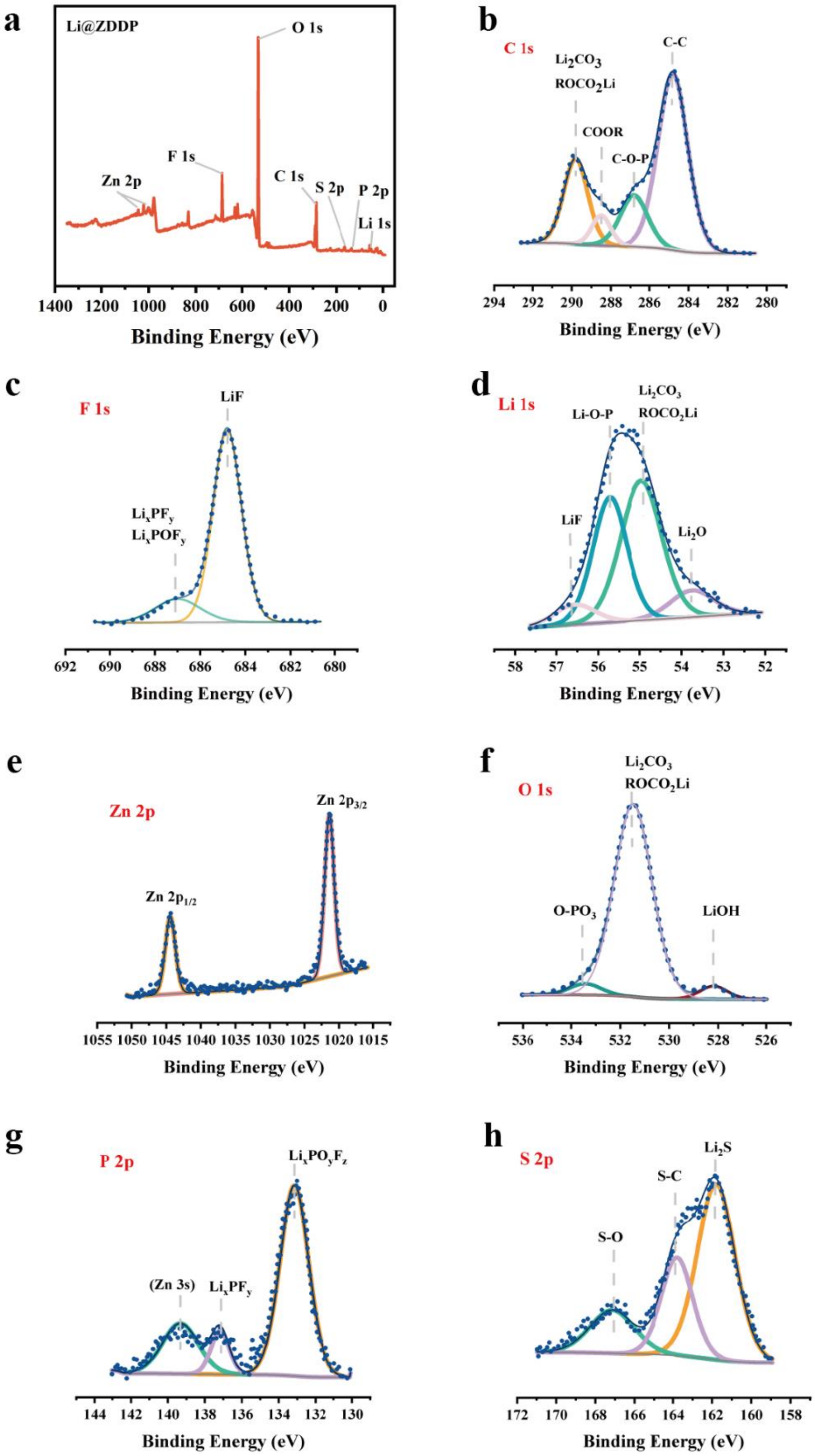

Figure S5. XPS spectra of Li@ZDDP electrode after 10 cycles. (a) Survey, (b) *C 1s* spectra, (c) *F 1s* spectra, (d) *Li 1s* spectra, (e) *Zn 2p* spectra, (f) *O 1s* spectra, (g) *P 2p* spectra, (h) *S 2p* spectra.

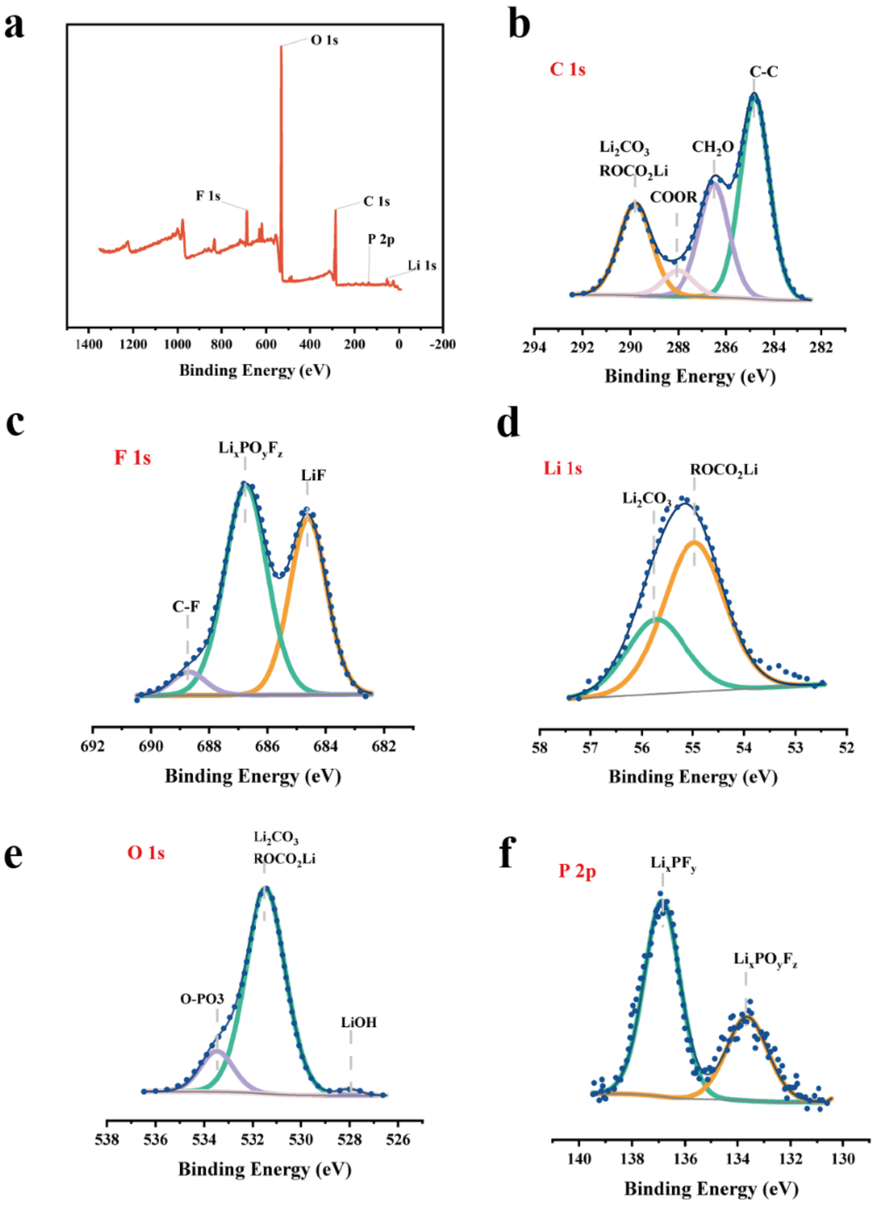

Figure S6. XPS spectra of Li electrode after 10 cycles. (a) Survey, (b) *C 1s* spectra, (c) *F 1s* spectra, (d) *Li 1s* spectra, (e) *O 1s* spectra, (f) *P 2p* spectra.

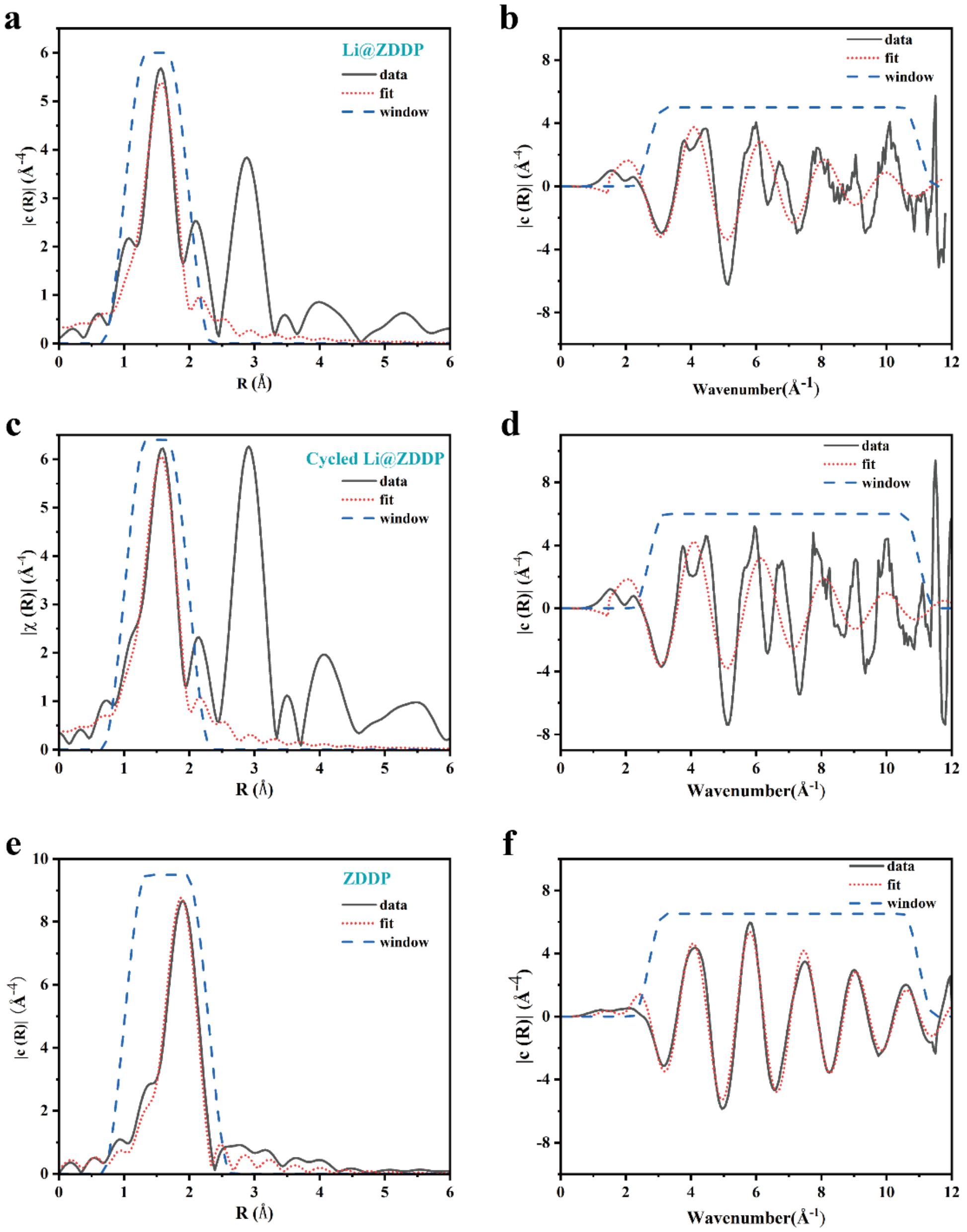

Figure S7. (a, b) Fitting results of k^3-weighted Zn K-edge EXAFS spectrum of Li@ZDDP plotted in R-space and k-space, respectively; (c, d) Fitting results of k^3-weighted Zn K-edge EXAFS spectrum of Cycled Li@ZDDP plotted in R-space and k-space, respectively; (e, f) Fitting results of k^3-weighted Zn K-edge EXAFS spectrum of ZDDP plotted in R-space and k-space, respectively.

a b

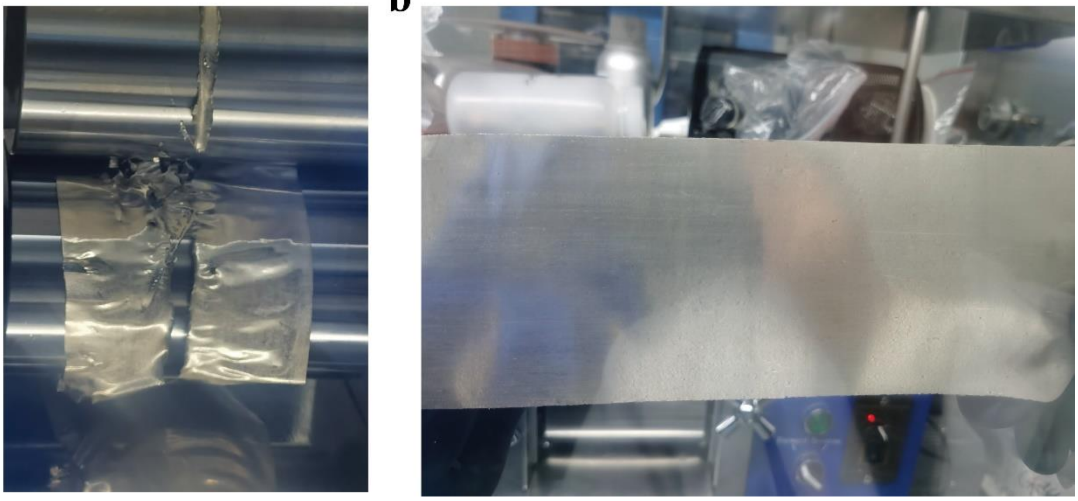

Figure S8. Optical photos of lithium strips (rolling to 40 μm) during actual processing. (a) No ZDDP was used for rolling (b) Rolling by using ZDDP

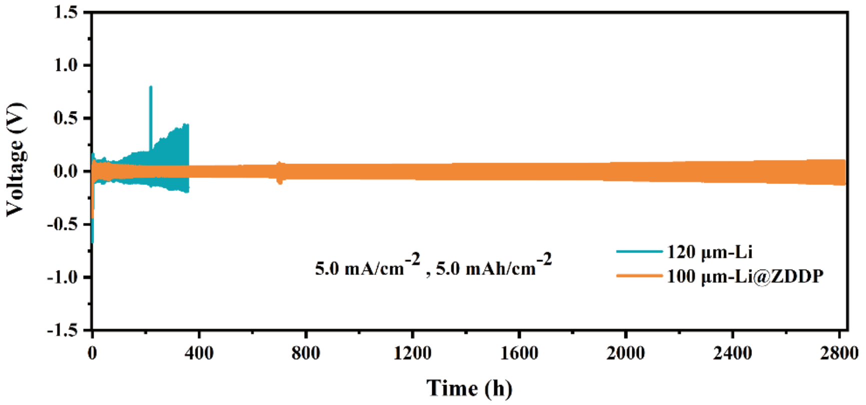

Figure S9. The voltage-time profiles of symmetrical cells with Li and Li@ZDDP at 5.0 mA cm^{-2} and 5.0 mA h cm^{-2}.

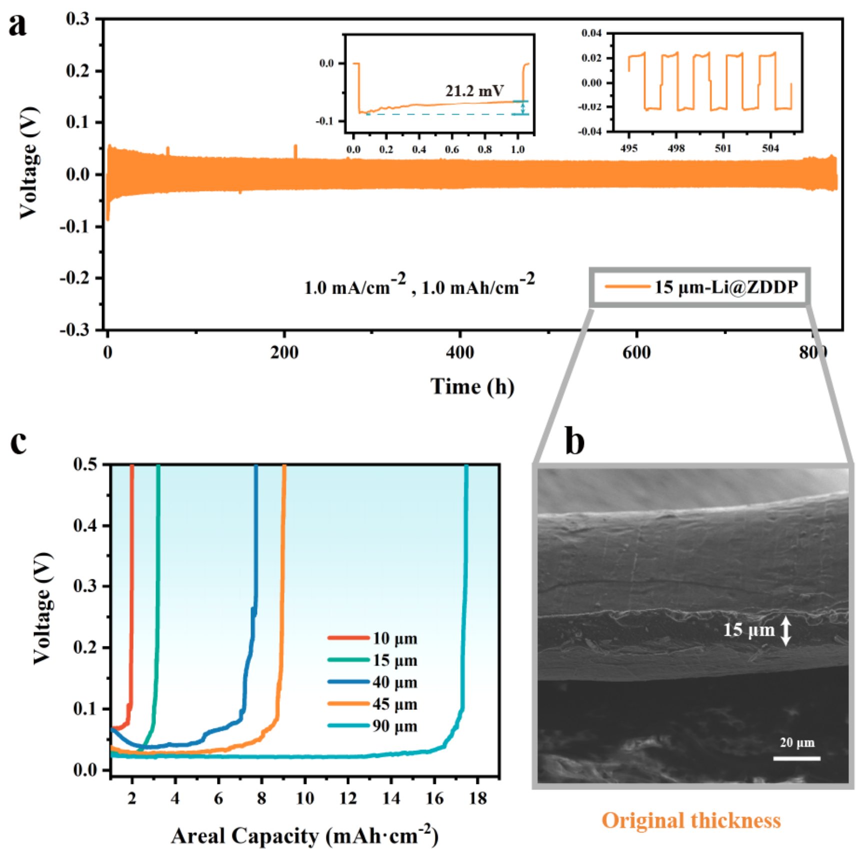

Figure S10. (a) Lithium stripping/plating cycling of symmetric cells with Li@ZDDP (15-μm-thick) electrodes at 1.0 mA cm^{-2} and 1.0 mA h cm^{-2} using 1 M LiTFSI in DME:DOL=1:1 Vol% with 4.0 M $LiNO_3$ electrolyte (LS020, Dodo Chem). (b) SEM image of 15-μm-thick electrodes before cycling. (c) Surface capacity of Li@ZDDP with different thicknesses.

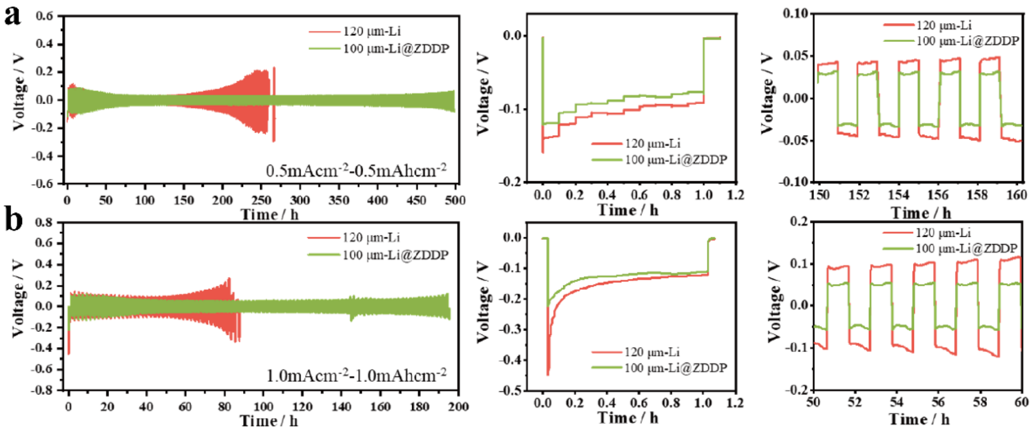

Figure S11. The voltage-time profiles of symmetrical cells with Li and Li@ZDDP at various current densities: (a) 0.5 mA cm^{-2} and 0.5 mA h cm^{-2}, (b) 1.0 mA cm^{-2} and 1.0 mA h cm^{-2}.

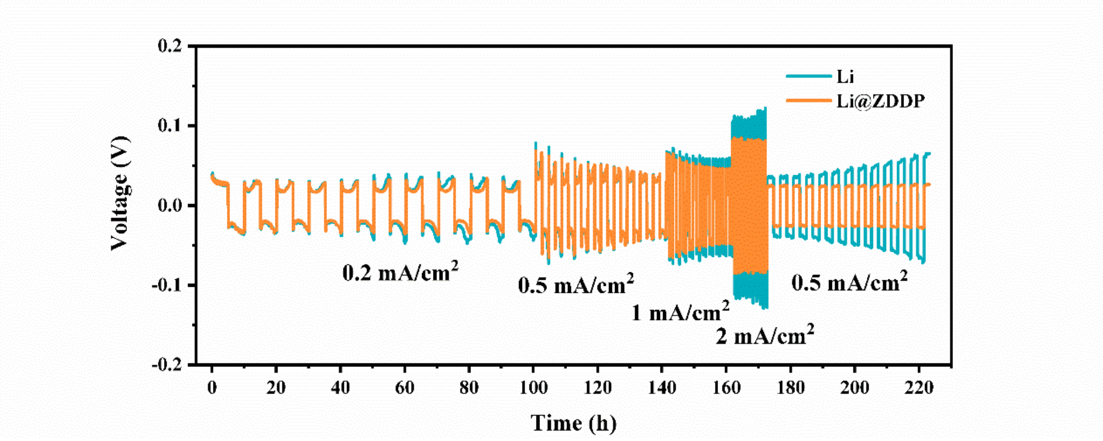

Figure S12. Rate performance of Li||Li and Li@ZDDP||Li@ZDDP symmetrical cell.

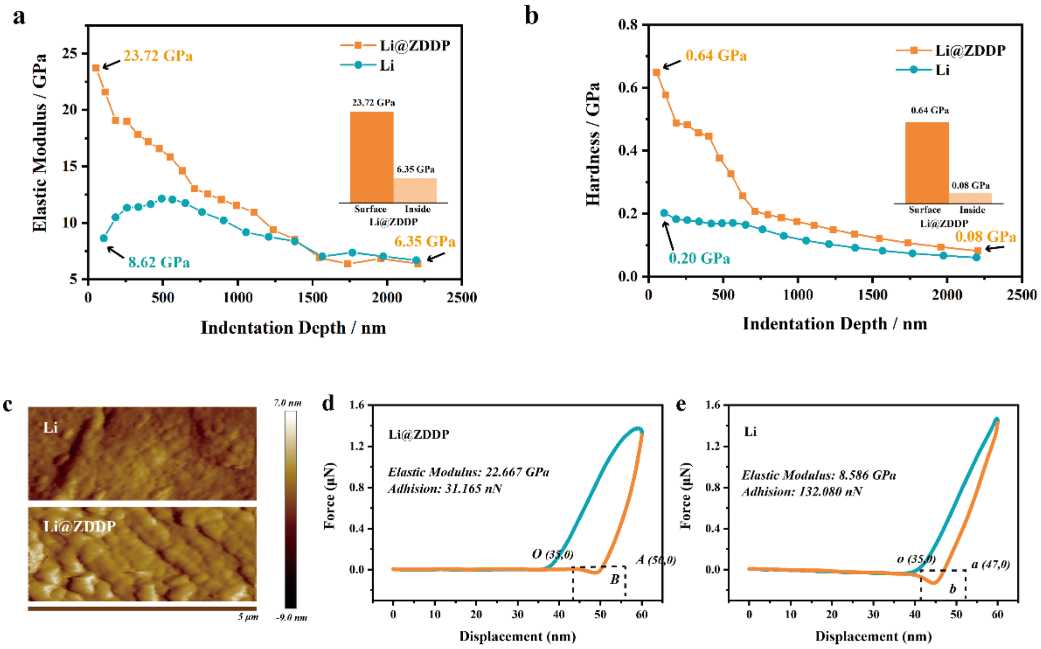

Figure S13. Comparison of elastic modulus (a) and hardness (b) between bare Li and Li@ZDDP using nanoindentation measurements. (c) Images of SEI on bare Li foil and Li@ZDDP after the charging–discharging cycle on AFM topography. The force–distance curves of the (d) SEI of Li@ZDDP after the charging–discharging cycle and (e) SEI of bare Li after the charging–discharging cycle.

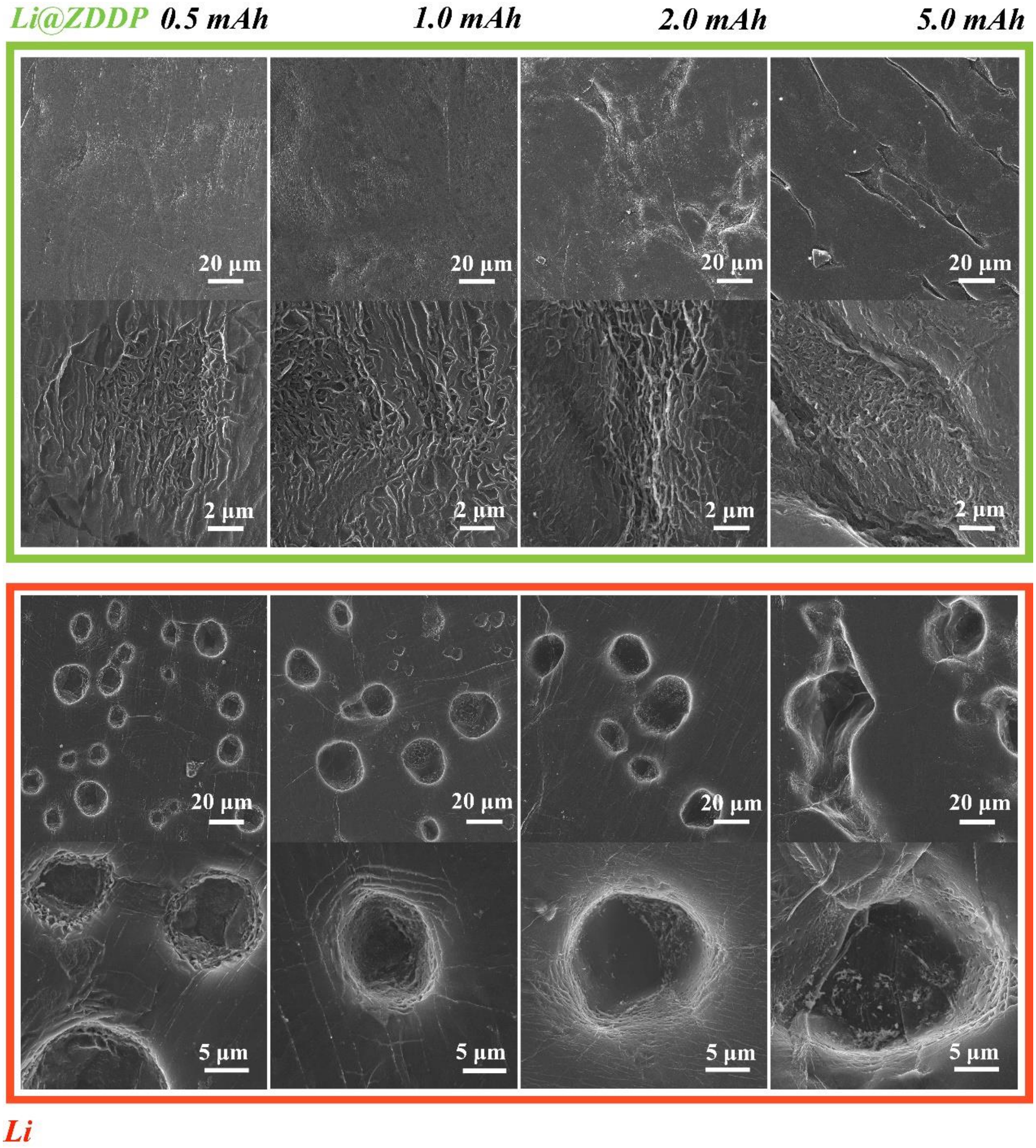

Figure S14. Morphology image of different capacity of the first Li stripping at 1.5 mA cm^{-2}.

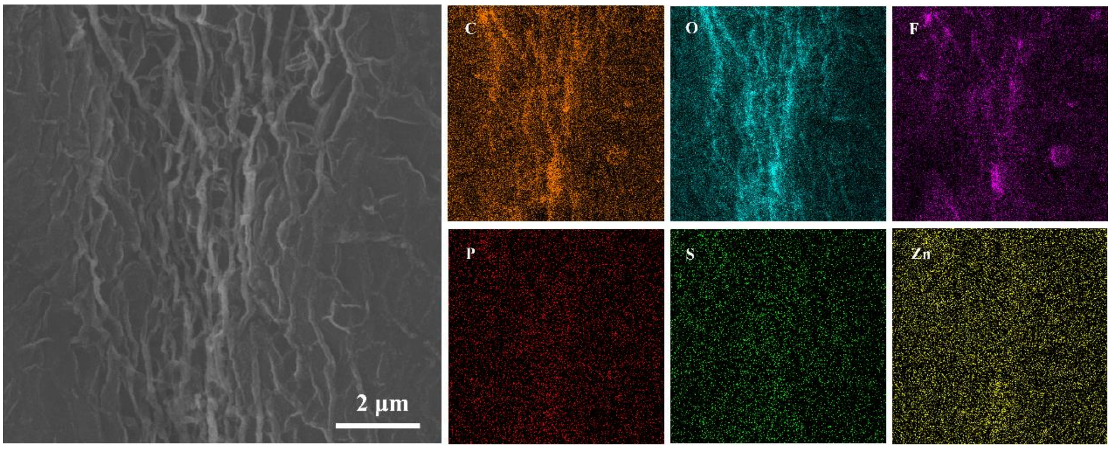

Figure S15. The EDS image of 2.0 mA h Li stripping at 1.5 mA cm^{-2}.

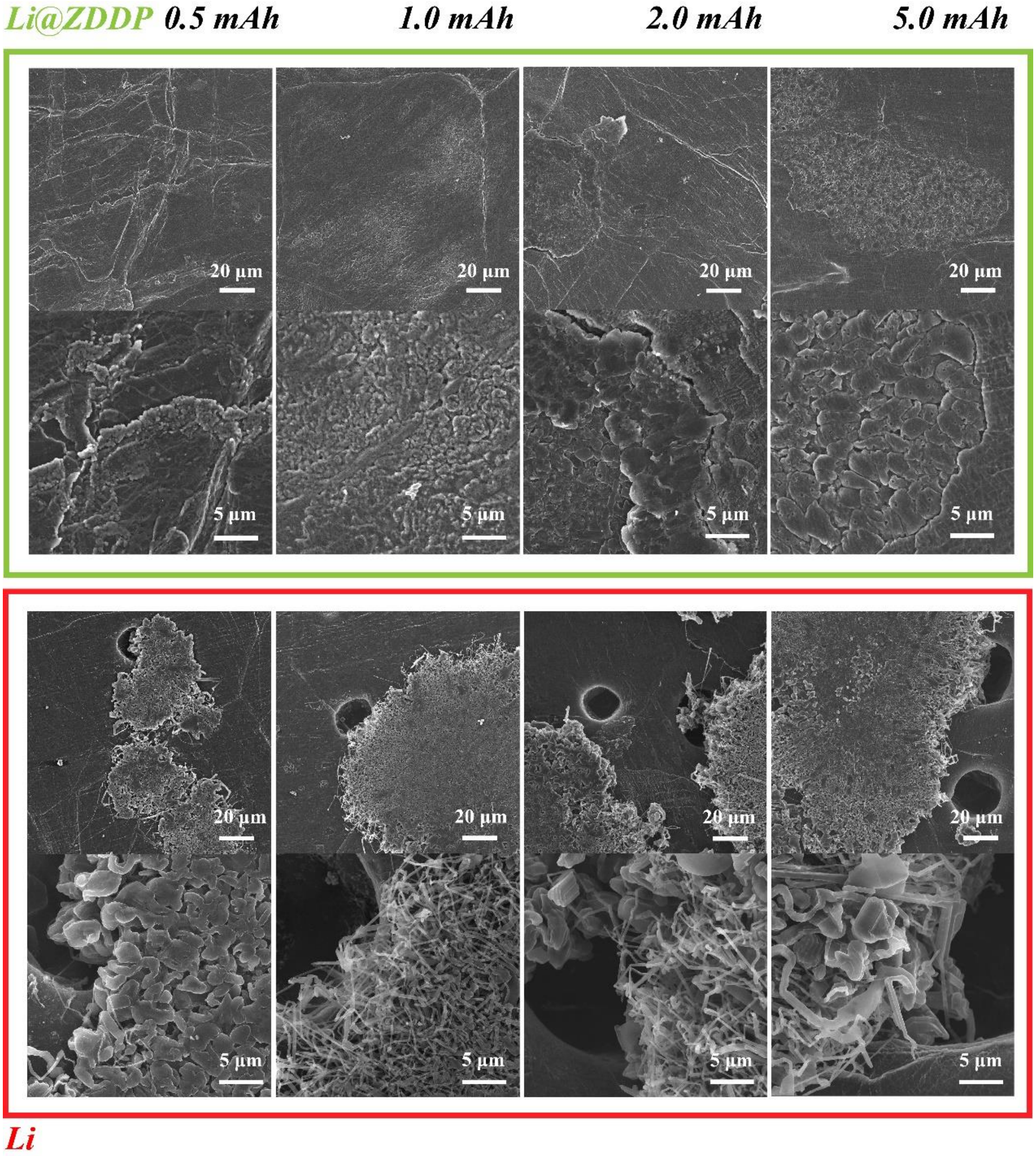

Figure S16. Morphology image of different capacity of the first Li plating after stripping at 1.5 mA cm^{-2}.

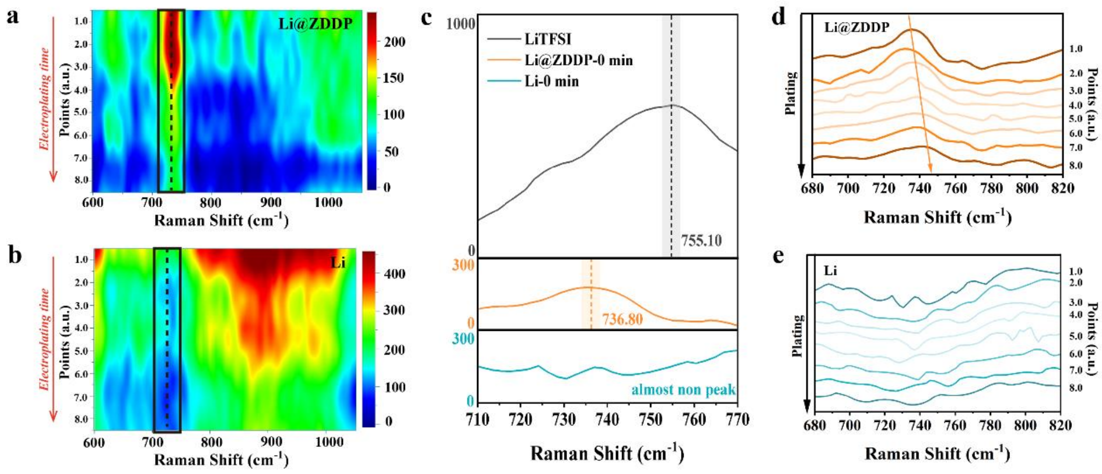

Figure S17. *In situ* Raman spectra of electrolyte near anode–electrolyte interface during Li@ZDDP (a, d) and Li plating (b, e) with 1.0 M LiTFSI in DME:DOL=1:1 Vol% at the plating current density of 3 mA cm^{-2}. (c) Initial Raman spectra of Li and Li@ZDDP near anode–electrolyte interface.

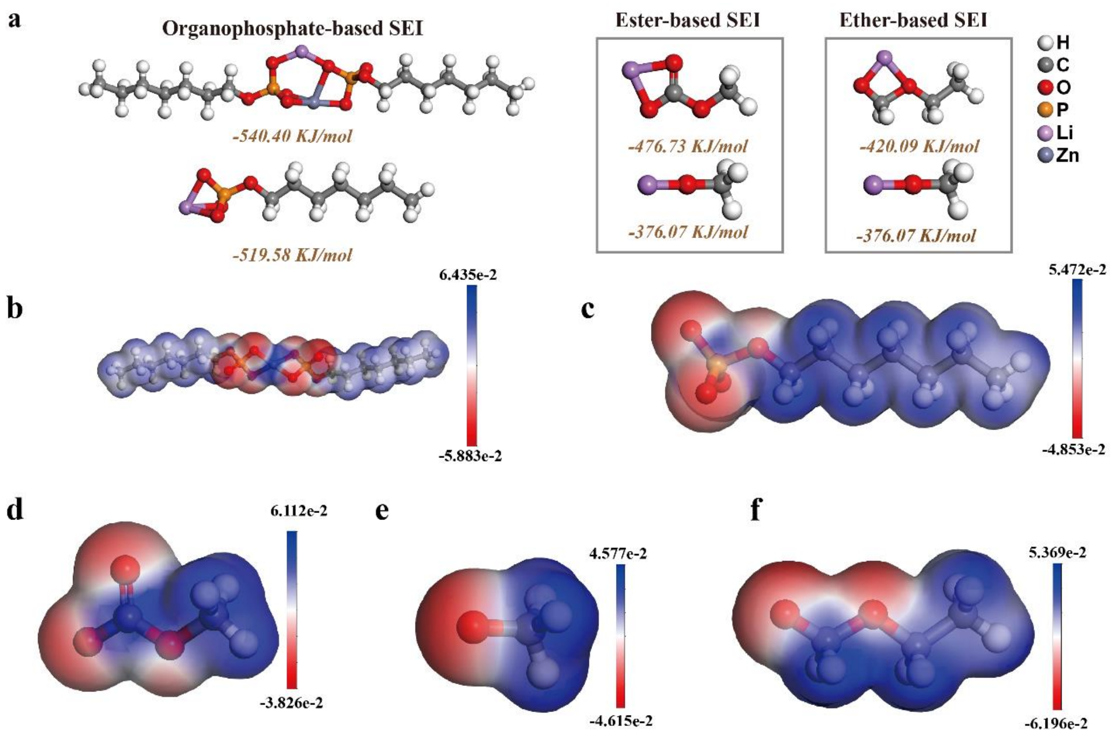

Figure S18. (a) Atomic structure and adsorption energy among Li+ and the nano bifunctional film (above), ester-based SEI (bottom left) and ether-based SEI (bottom right). (b-f) The calculated electrostatic potential (ESP).

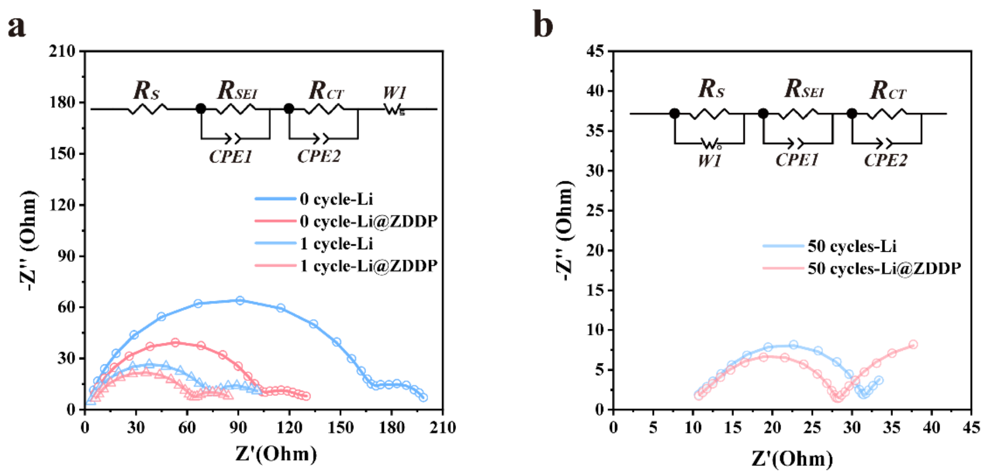

Figure S19. EIS test of Li||Li and Li@ZDDP||Li@ZDDP symmetrical cell at 18 mA cm^{-2} and 1.5 mA cm^{-2} after (a) 0 cycle, 1 cycle and (b) 50 cycles at 25 ℃.

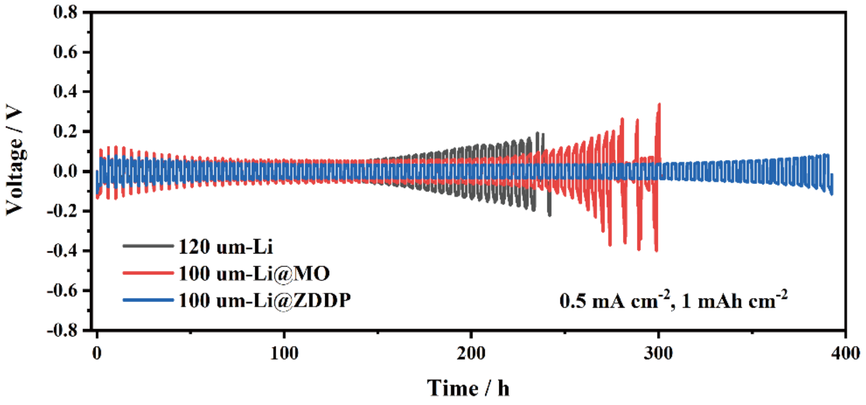

Figure S20. The voltage-time profiles of symmetrical cells with Li, Li@MO and Li@ZDDP at 5.0 mA cm^{-2} and 5.0 mA h cm^{-2} using 1.0M $LiPF_6$ in EC:DMC:DEC=1:1:1 Vol% with 1.0%VC as electrolyte.

Table S1 Zn K-edge EXAFS curves fitting parameters.

Sample	Path	*N*	*R(Å)*	*ΔR(Å)*	σ^2×10^{-3}(Å^2)	ΔE_0 (eV)	R-factor (%)
**Li@ZDDP**	Zn-O	3.6±0.6	2.01	0.08±0.02	10±3	8.07±1.96	1.30%
**Cycled Li@ZDDP**	Zn-O	4.1±0.4	2.01	0.08±0.01	10±2	8.04±1.14	0.42%
**ZDDP**	Zn-S	4.0±0.5	2.33	0.01±0.01	9±2	2.21±1.18	0.93%

N, coordination number; *R*, coordination distance between absorber and backscatter atoms; σ^2, Debye–Waller factor to evaluate both thermal and structural disorders; ΔE_0, inner potential correction; *R-factor* indicates the goodness of the fit. S_0^2 was fixed to 1.0 as determined from the fit of Zn foil and ZnO reference. The error bars were obtained from running the Artemis program with the EXAFS fitting.

Table S2 EIS curves fitting parameters.

Sample	R_s	Error-R_s (%)	R_{SEI}	Error- R_{SEI} (%)	R_{CT}	Error- R_{CT} (%)
0 cycle-Li	0.94	8.09	168.50	0.40	32.33	5.08
0 cycle-Li@ZDDP	2.32	2.95	101.50	0.54	21.64	26.63
1 cycle-Li	1.17	6.18	71.63	2.52	30.72	22.04
1 cycle-Li@ZDDP	2.97	5.12	61.19	1.59	19.36	55.98
50 cycles-Li	12.11	2.28	19.48	1.73	12.48	23.74
50 cycles-Li@ZDDP	11.96	2.15	16.07	1.83	26.33	5.34

R_S, Ohmic Impedance; R_{SEI}, Li^+ transfer resistance; $R_{CT,}$ charge transfer resistance. The error bars were obtained from running the ZVIEW program with the EIS fitting. Prioritize to control of the error- R_{SEI} in fitting analysis.
