# Supplementary figures and images for: Interfacial friction enabling ≤ 20 μm thin free-standing lithium strips for lithium metal batteries
Source: Nat Commun. 2023 Sep 14;14:5678. doi: 10.1038/s41467-023-41514-0 (PMC10502130; doi:10.1038/s41467-023-41514-0)

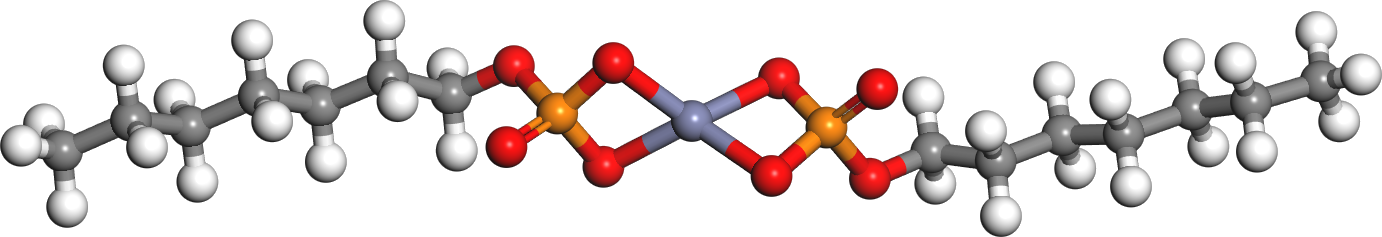

Supplement: Supplementary file 3 — Source Data [file 41467_2023_41514_MOESM3_ESM.zip › Source Data/Supplementary Figure S18-DFT/C14P2O8/C14P2O8.bmp]

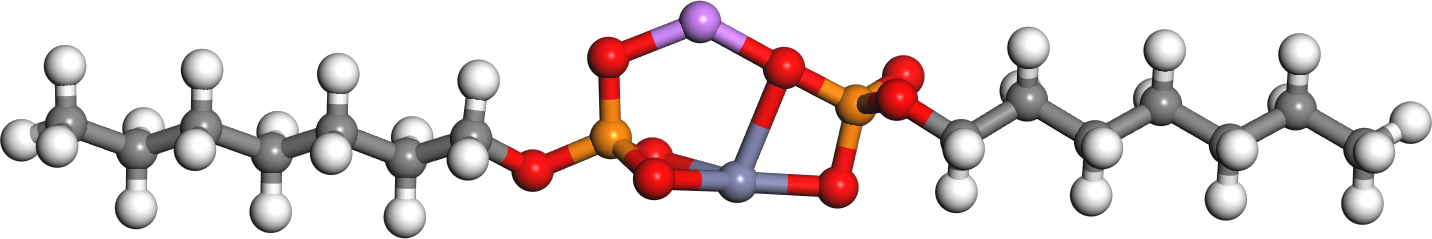

Supplement: Supplementary file 3 — Source Data [file 41467_2023_41514_MOESM3_ESM.zip › Source Data/Supplementary Figure S18-DFT/C14P2O8/C14P2O8-Li.bmp]

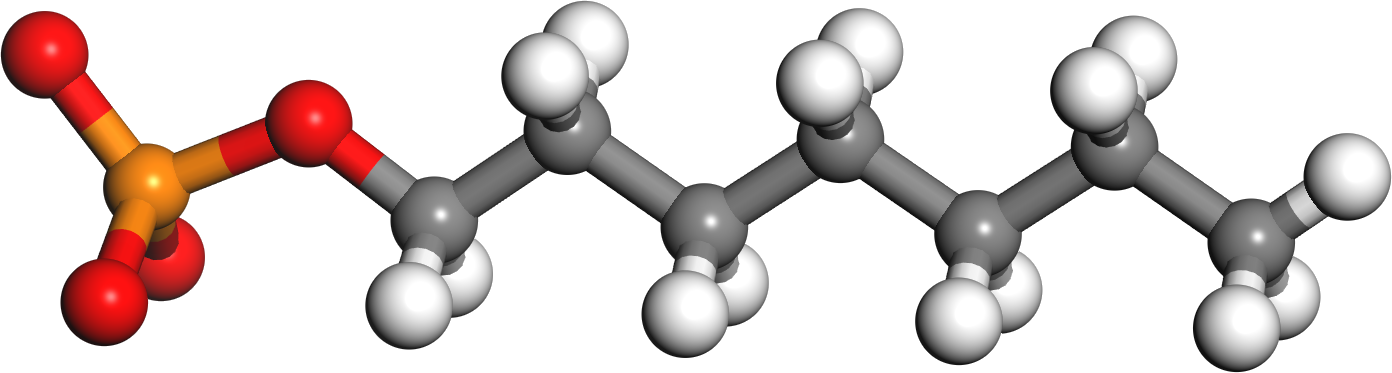

Supplement: Supplementary file 3 — Source Data [file 41467_2023_41514_MOESM3_ESM.zip › Source Data/Supplementary Figure S18-DFT/C7PO4/C7PO4.bmp]

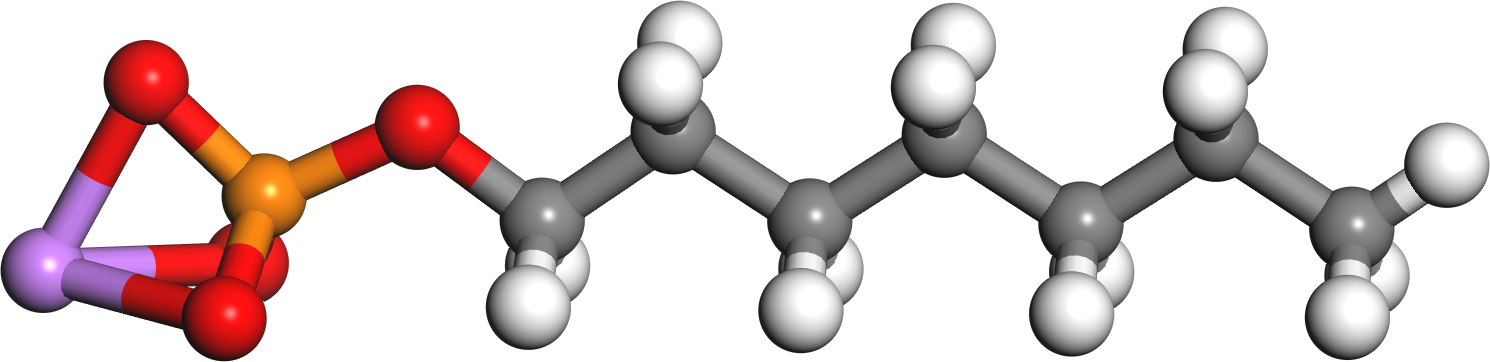

Supplement: Supplementary file 3 — Source Data [file 41467_2023_41514_MOESM3_ESM.zip › Source Data/Supplementary Figure S18-DFT/C7PO4/C7PO4-Li.bmp]

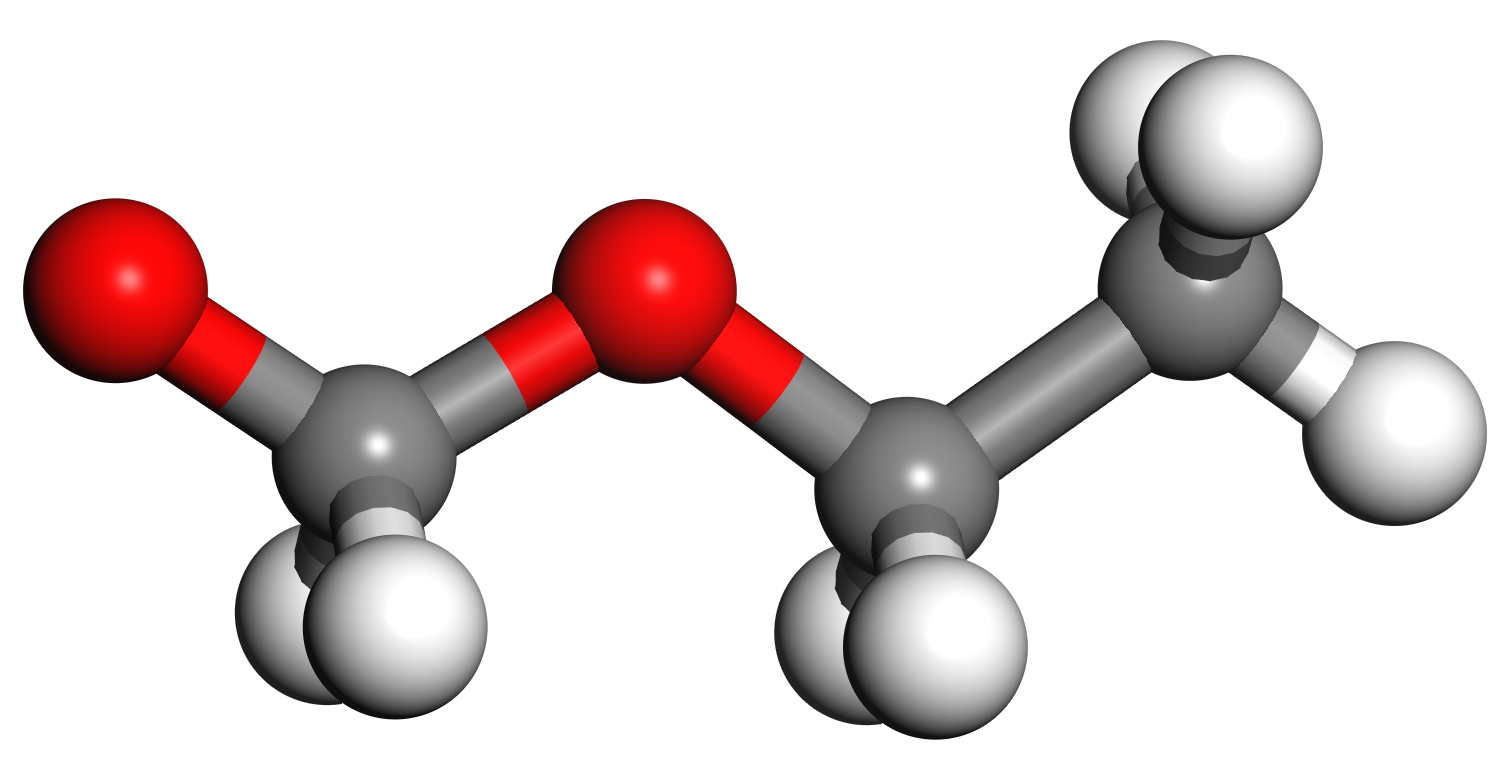

Supplement: Supplementary file 3 — Source Data [file 41467_2023_41514_MOESM3_ESM.zip › Source Data/Supplementary Figure S18-DFT/CH2O2CH2CH3/CH2O2CH2CH3.bmp]

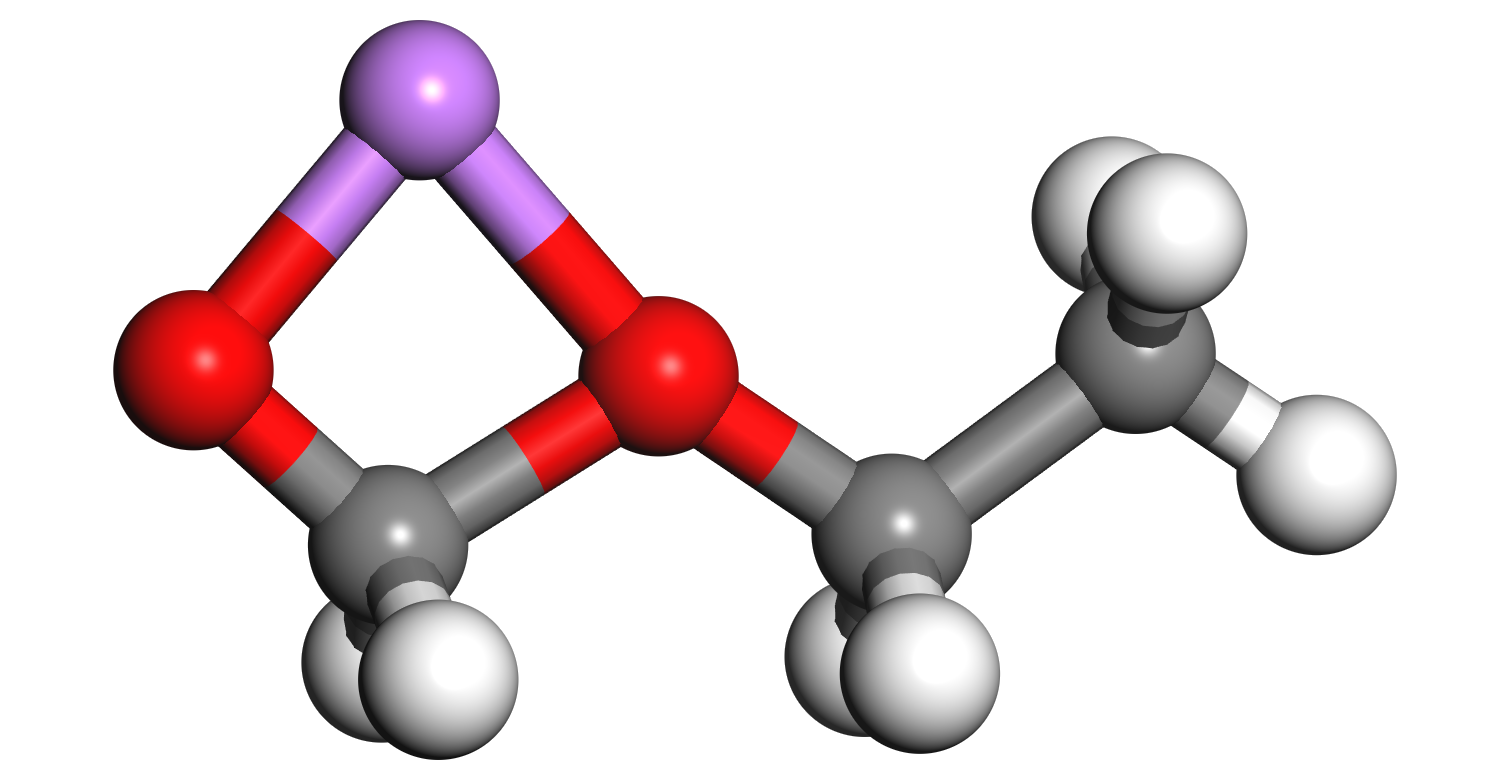

Supplement: Supplementary file 3 — Source Data [file 41467_2023_41514_MOESM3_ESM.zip › Source Data/Supplementary Figure S18-DFT/CH2O2CH2CH3/CH2O2CH2CH3-Li.bmp]

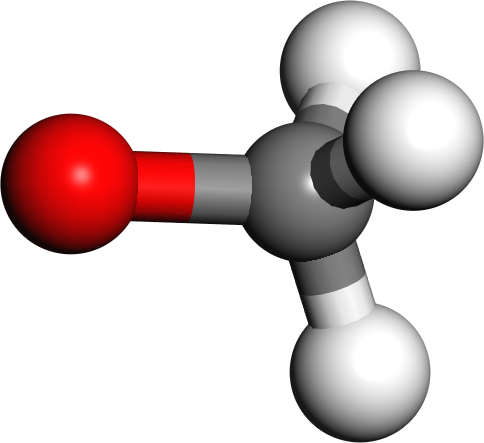

Supplement: Supplementary file 3 — Source Data [file 41467_2023_41514_MOESM3_ESM.zip › Source Data/Supplementary Figure S18-DFT/CH3O/CH3O.bmp]

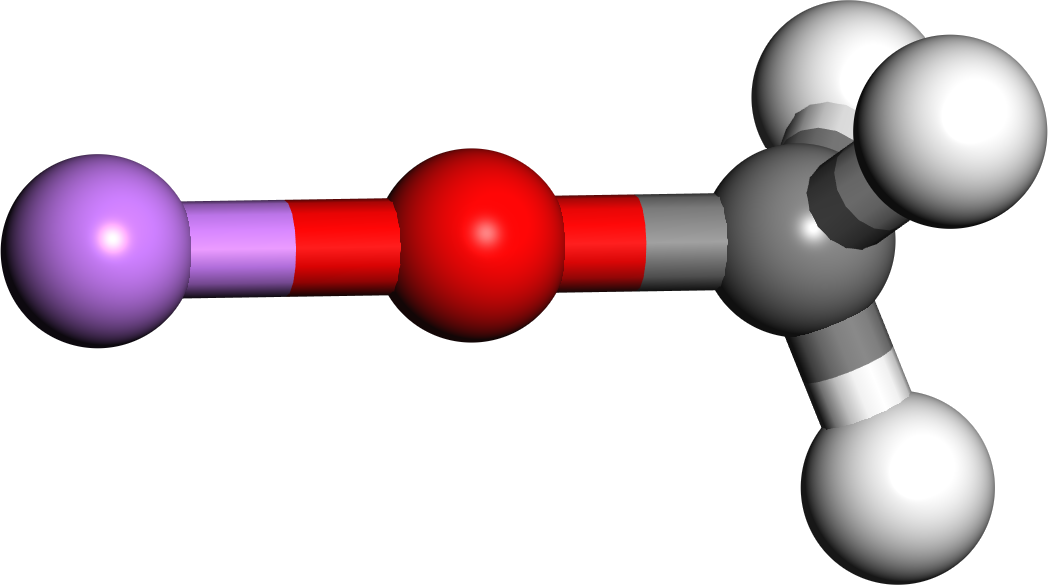

Supplement: Supplementary file 3 — Source Data [file 41467_2023_41514_MOESM3_ESM.zip › Source Data/Supplementary Figure S18-DFT/CH3O/CH3O-Li.bmp]

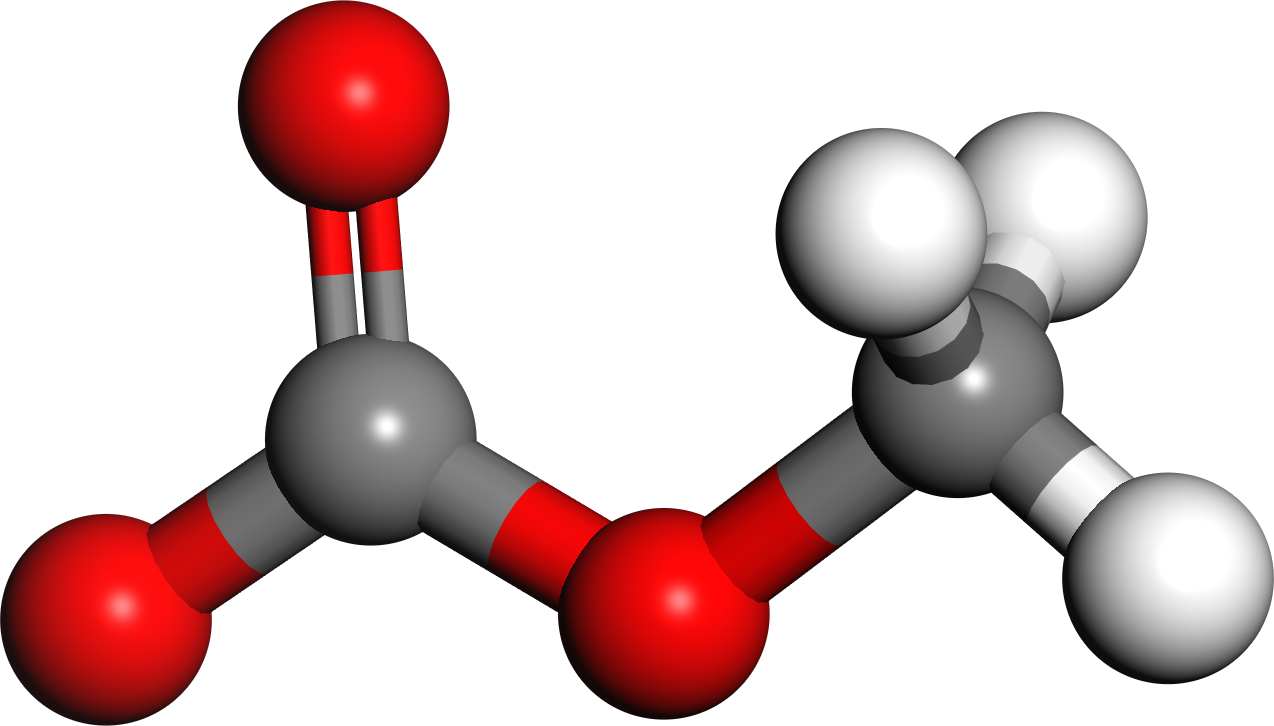

Supplement: Supplementary file 3 — Source Data [file 41467_2023_41514_MOESM3_ESM.zip › Source Data/Supplementary Figure S18-DFT/CO3CH3/CO3CH3.bmp]

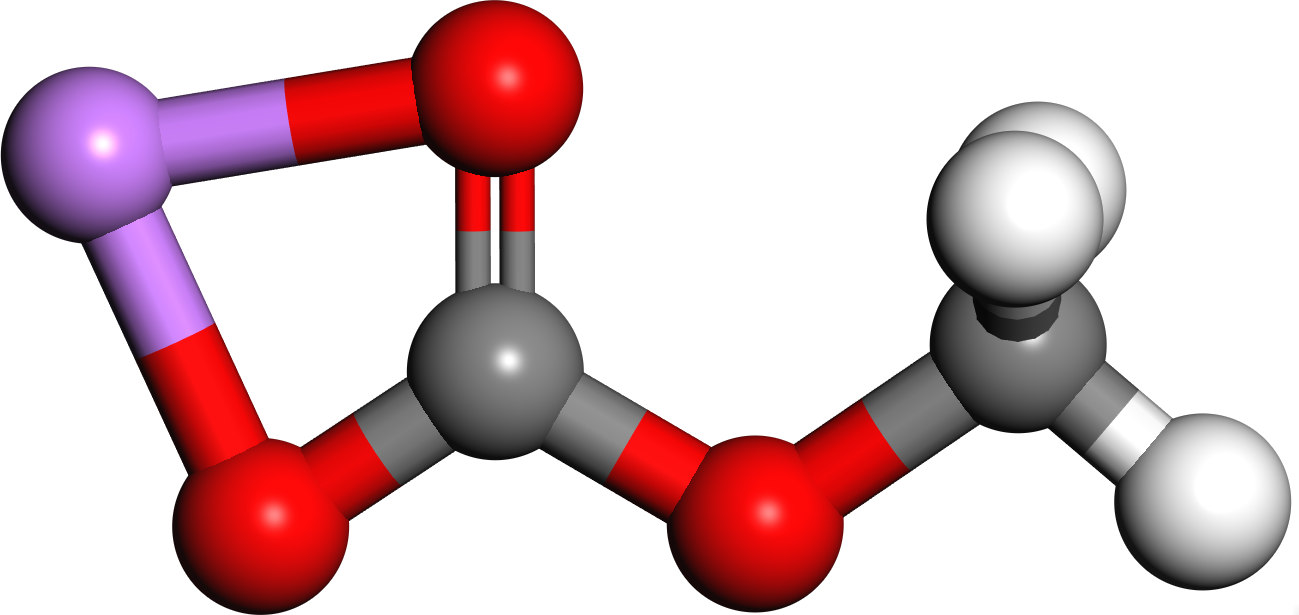

Supplement: Supplementary file 3 — Source Data [file 41467_2023_41514_MOESM3_ESM.zip › Source Data/Supplementary Figure S18-DFT/CO3CH3/CO3CH3-Li.bmp]
